# Sunflower-based Feedstocks in Nonfood Applications: Perspectives from Olefin Metathesis

**DOI:** 10.3390/ijms9081393

**Published:** 2008-08-13

**Authors:** Bassie B. Marvey

**Affiliations:** Department of Chemistry, North-West University, P/Bag X2046, Mafikeng, 2735, South Africa. Tel. +27-18-389-2527; Fax: +27-18-389-2052; E-Mail: bassie.marvey@nwu.ac.za

**Keywords:** sunflower, nonfood applications, olefin metathesis

## Abstract

Sunflower (*Helianthus annuus L*.) oil remains under-utilised albeit one of the major seed oils produced world-wide. Moreover, the high oleic sunflower varieties make the oil attractive for applications requiring high temperature processes and those targeting the C=C double bond functionality. Herein an overview of the recent developments in olefin metathesis of sunflower-based feedstocks is presented. The improved performance of olefin metathesis catalysts leading to high turnover numbers, high selectivity and catalyst recyclability, opens new opportunities for tailoring sunflower-based feedstocks into products required for possible new niche market applications. Promising results in biofuel, biopolymers, fragrances and fine chemicals applications have been reported.

## 1. Introduction

The rapid depletion of crude oil reserves, escalating fuel prices and the concern over climate change catapulted into global interest for utilizing crop-based feedstocks as renewable alternatives to petroleum-based feedstocks. According to some analysts [1] oil production levels should hit their maximum soon after 2010 thus causing prices of petrol and other fuels to reach disastrous levels. For this reason non-fossil fuels must phase in much faster than it had been hoped. Crop-based feedstocks have the advantage over petroleum-based ones in that the resources are renewable, the products are biodegradable and their processes result in reduced emissions of greenhouse and harmful gases [2]. This has subsequently led to much interest in nonfood-applications of seed oils to compensate for the limited supply and the escalating price of petroleum and petroleum-based products [3]. However, there are few limitations with natural oils still to be overcome, namely, the fact that in major seed oils such as sunflower, rapeseed, soybean and palm oil the main fatty acids have the carbon chain length ranging from C16 to C18 and lack functionalities that give them wider industrial application such as the hydroxy and the epoxy groups [4]. The challenge, then, is for scientists and engineers to find ways of improving on the fatty acid composition of these oils for niche markets and to develop processes for converting these alternative feedstocks to high value intermediates and finished products [5].

Already, biochemical and genetic studies are revealing pathways of fatty acid biosynthesis in oilseeds which could be exploited for modifying the existing seed oils to make them more industrially attractive [5]. Meanwhile, progress is being made with regard to the variation of the carbon chain length of these oleochemicals and the introduction of important functionalities using chemical manipulation methods [6–9]. Olefin metathesis, for example, has become a useful synthetic tool by which seed oil-derived olefins could be converted in relatively clean catalytic processes to value-added products. Recent improvements in catalyst design and performance (especially on Grubbs and Hoveyda-type catalysts) have opened new opportunities for application of functionalized olefins in cross-metathesis, ring closing metathesis and materials synthesis [10–13]. Presented in this paper is an overview of the developments in olefin metathesis involving sunflower-based feedstocks and their potential application as raw materials for the chemical industry.

## 2. Sunflower: Description and Oil Characteristics

Sunflower crop (*Helianthus annuus L*.), originally of subtropical and temperate zones [14], is widely adaptable and more drought tolerant than most other grain crops [15]. It grows best on soil with a high water-holding capacity but is easily adapted to a range of soil conditions [14]. The estimated global sunflower seed output is around 25 million tons versus the global sunflower oil output estimated at 10 million tons produced from over 80% of the global sunflower seed production [16]. The average oil content of the seed is 40–50% [14] and according to Campbell [17], linoleic/oleic distribution in sunflower oil is mostly affected by the growing temperatures with cooler climates favouring linoleic acid. Nowadays natural plant breeding methods have enhanced the fatty acid profile of the oil by making it higher in oleic acid [18]. The three types of sunflower oil now available on the market are NuSun, high oleic and regular (linoleic) sunflower oil. NuSun is a mid-oleic form with 65 percent oleic acid; high oleic sunflower oil is 80 percent oleic acid and regular sunflower oil is 69 percent linoleic acid [19, 20]. High levels of oleic acid make the oil more resistant to oxidation and hence more suitable for processes requiring high oxidative stability at high temperatures as in biodiesel and biolubricants applications [21, 22]. On the other hand, high linoleic acid content (less stable to oxidation) makes the oil to have excellent drying properties, which is a desirable property for applications in paints, inks and varnishes.

## 3. Nonfood Applications Based On Olefin Metathesis

Recent developments in olefin metathesis offer new opportunities for converting crop-based feedstocks, especially vegetable oils, into a wide range of high-value products required for possible new niche market applications. In the European Union (EU-15) alone, for example, the nonfood sector consumes about 3 million tons of vegetable oil per annum with the exclusion of biodiesel [23]. Most of this oil is used in commodities such as paints, surface coatings, lubricants, surfactants and oleochemicals. Sunflower oil is mostly used as a smoothing agent in cosmetics and skin creams. There are also opportunities in the biofuel, polymer, surfactant, flavour and fragrance industries. The production of hydrogen from sunflower oil for use in fuel cells (that would power the fuel-cell motor) has also been reported [24]. According to Dupont [24], hydrogen has a very high energy content compared to natural gas and coal and stands as a promising candidate for renewable clean energy technologies. Discussed in subsequent sections are some of the potential industrial applications in which the olefin metathesis reaction is employed as part of the production process.

### 3.1. Biofuel

**Scheme 1:**
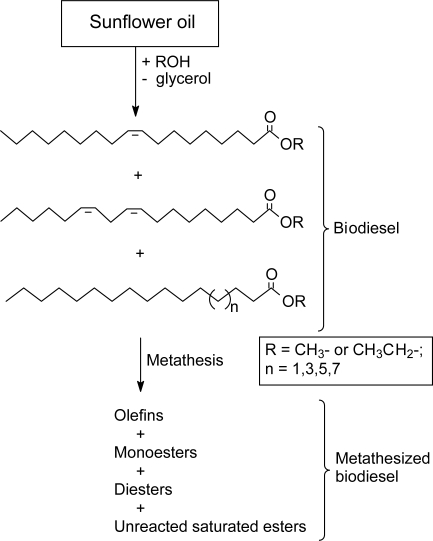
Transesterification of sunflower oil and modification by olefin metathesis.

Sunflower oil is ranked the second after rapeseed oil for biodiesel use in Europe [25]. The other major biodiesel seed oils are soybean and palm oils with jatropha oil being amongst the highly promising biodiesel feedstocks. The oleic acid content in high oleic sunflower oil makes the oil more stable to oxidation and more suited for biofuel production [26]. As illustrated in scheme 1, biodiesel is derived from natural oils *via* alcoholysis, a transesterification reaction. The lower alcohol such as methanol or ethanol is commonly employed for the alcoholysis process. Biodiesel is a very light oil, less viscous, very lubricating and can be used in diesel engines including trucks, generators, boats, trains, busses and cars [27]. Glycerol, the by-product of transesterification, is on the other hand an attractive starting material for the synthesis of propylene glycol, 1,3-propanediol and epichlorohydrin, which are all important industrial monomers [28]. This glycerol market should help offset some of the costs involved in producing biodiesel.

Of importance are the combustion and flow properties of this type of fuel which are determined primarily by the fatty acid content of the vegetable oil [29, 30]. Fuel properties of selected biodiesels are depicted in Table 1. A diesel with a cetane number (CN) between 40 and 50 is recommended for diesel engines. Fuel with a higher CN is preferred due to its shorter ignition delay time. In North America regular diesel has a CN of 40–46 and 45–50 for premium diesel [31]. Tests done in the US and Europe have shown that engines running on biodiesel have minor, if any differences, in torque, horse power, range, and top speed to those running on petroleum-based diesel [2].

Although neat biodiesel (B100) could be used in most diesel engines directly, the fuel is more commonly blended with petroleum diesel in varying proportions, with the most common diesel mix being B2 (2% biodiesel) and B20 (20% biodisel) [32]. On the other hand, few chemical modifications could bring some improvements on its performance properties. One such modification involves the cross metathesis of the olefinic fatty acid esters (essentially oleic and linoleic acid esters) resulting in a mixture of olefins, monoesters and diesters as shown in Scheme 1. To achieve this kind of fuel reformulation a number of catalytic systems based on W, Re and Ru have proved effective (Table 2). These catalysts act selectively at the unsaturated sites of the olefinic fatty esters with minimal activity for the substituent groups. The saturated esters remain essentially unaffected due to the absence of carbon-carbon double bonds.

Nicolaides *et al.* [34] reported the metathesis of sunflower-based biodiesel (ethyl esters) using a homogeneous WCl_6_/SnMe_4_ catalyst. In our laboratory [35] we performed the metathesis of sunflower-based biodiesel (methyl esters) using the heterogeneous 3% Re_2_O_7_/SiO_2_−Al_2_O_3_/SnBu_4_ catalyst. The tungsten and rhenium based catalytic systems gave comparably high conversions with a high selectivity. The products (olefins, mono- and diesters) were the result of the cross metathesis between oleic and linoleic acid esters as illustrated in Scheme 1. According to Holser *et al.* [36] a reformulation of the fuel by olefin metathesis is expected to improve the viscosity and lubricity of biodiesel without decreasing storage or adversely affecting its biodegradability. The high viscosity of vegetable oils is known to result in poor atomization in the combustion chamber and has been identified as a major cause of nozzle coking and engine deposits [37, 38]. Due to the significance of viscosity as one of the properties influencing biodiesel quality, convenient methods have been developed by Allen *et al.* [39] and Shu *et al.* [40] which correlate viscosity to biodiesel components. Any of these methods should eliminate the need for expensive measurements and could be used to select feedstock that will appropriately produce biodiesel with desired specifications. The reader is referred to an article by Knothe *et al.* [33] on issues of economics, regulations, combustion chemistry and suitability of biodiesel for conventional diesel engines.

### 3.2. Polymers

**Scheme 2:**
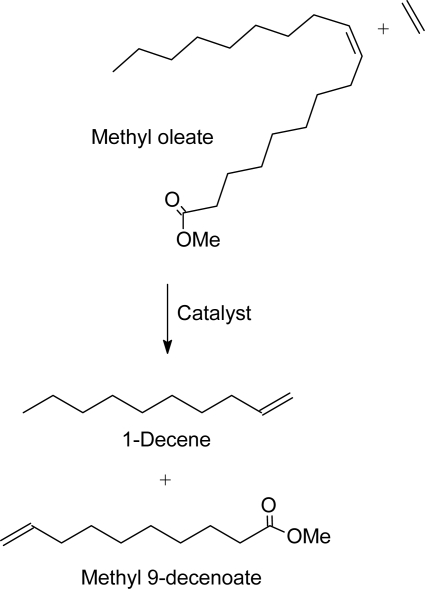
Ethenolysis of methyl oleate.

The chemical industry is already showing keen interest in nonfood applications of seed oils using olefin metathesis technology. At Dow [41], for example, the development of olefin metathesis catalysts for the ethenolysis of methyl oleate (Scheme 2) has been identified as critical for the conversion of seed oil-based feedstocks into useful raw materials for the synthesis of niche market materials such as epoxy thermoplastics and thermosets, polyurethane foams, thermoplastic polyurethanes, polyolefin comonomers and surfactants. Methyl oleate is the main constituent in high oleic sunflower oil. Thanks to the ground-breaking work of Boelhouwer and coworkers. In 1972 the first homogeneously catalyzed self-metathesis of methyl oleate using WCl_6_/SnMe_4_ was reported [42]. Later on the heterogeneous Re_2_O_7_−Al_2_O_3_/SnMe_4_ was reported to catalyze the metathesis of unsaturated fatty acid esters with high selectivity (>99%) [43]. Since then, attempts have been made to improve the stability and the overall performance of tungsten and rhenium based catalysts or alternatively to develop new highly active, selective and stable catalytic systems. Another ground-breaking work was the discovery by the group of Grubbs [44–47] of the well-defined ruthenium-based catalysts, especially the well known Grubbs first generation catalyst RuCl_2_(PCy_3_)_2_(=CHPh) (**1**) which exhibits high activity and high tolerance towards a wide range of functional groups.

**Scheme 3:**
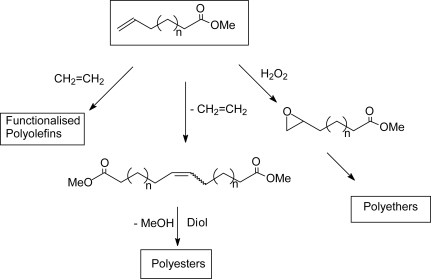
Polymers based on ω-unsaturated fatty acid methyl esters.

Warwel’s group [8] reported the synthesis of polyesters, functionalized polyolefins and polyethers starting with ω-unsaturated fatty acid methyl esters (FAMEs) as shown in Scheme 3. The latter were obtained from plant oils by ethenolysis using **1** as catalyst (Scheme 2). Oleic acid methyl ester was obtained from high oleic sunflower and rapeseed oils and other FAMEs from oils of coriander (petroselinic acid, 75%), meadowfoam (5-eicosenoic acid, 63%), high erucicrapeseed (erucic acid, 48%) and crambe (erucic acid, 59%).

**Scheme 4:**
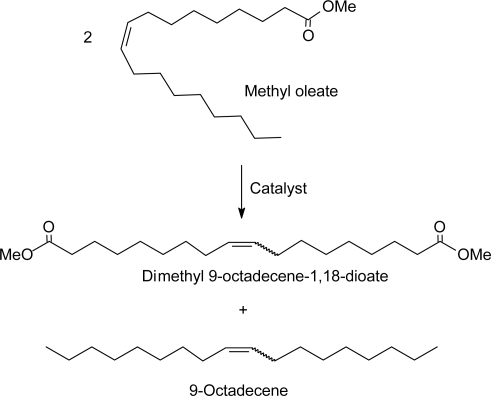
Self-metathesis of methyl oleate.

The self-metathesis of ω-unsaturated FAMEs using **1** led to the formation of dimers (α,ω-dicarboxylic acid methyl esters) which were subsequently polycondensated with diols to give polyesters [8, 48]. Functionalized polyolefins were produced by copolymerization of ethene with ωunsaturated FAMEs using the cationic palladium complex catalyst {[(2,6-*i*-PrPh)_2_DABMe_2_]Pd[(CH_2_)_3_COOMe]}SbF_6_ [49] and polyethers by epoxidation of ω-unsaturated FAMEs followed by ring-opening polymerization. Dimers can also be synthesized by the self-metathesis of methyl oleate (Scheme 4) although due to equilibrium limitations lower yields have been obtained in the presence of WCl_6_−SnMe_3_ [50,51], Re_2_O_7_−Al_2_O_3_/SnMe_4_ [43] and **1** [52]. However, Dinger and Mol [53] reported very high effective turnover numbers (440 000) and a high selectivity for the self-metathesis of methyl oleate in the presence of Grubbs second generation catalyst RuCl_2_(PCy_3_)(H_2_IMes)(=CHPh) (**2**). Cross-metathesis of methyl oleate with methyl acrylate is another way of preparing α,ω-dicarboxylic acid esters. Promising results have been obtained in this regard by Rybak and Meier [54] using Grubbs catalysts **1** and **2** as well as Hoveyda-Grubbs second generation catalyst **3**. Indeed, the highly efficient ruthenium catalysts opens new possibilities for introducing into the carbon chain structure new functionalities which would, otherwise, be difficult to achieve using conventional methods [55].

**Figure 1:**
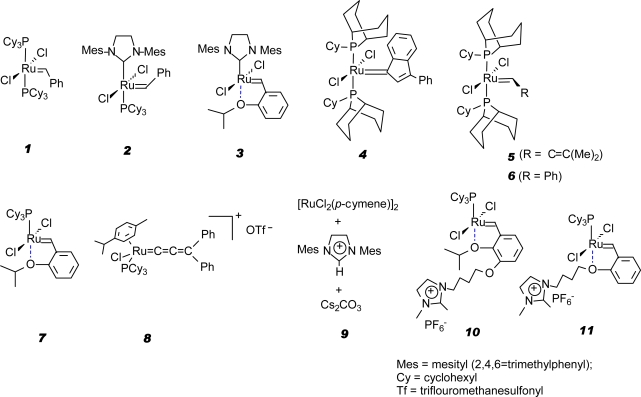
Ruthenium catalysts for olefin metathesis.

Attempts to improve the stability and the overall performance of the ruthenium-based catalysts led to the synthesis of a phoban-indenylidene ruthenium catalyst [(PhobCy)_2_Cl_2_Ru=C_15_H_10_] (**4**) by the Sasol group [56]. Improved turnovers and selectivity were obtained for the self-metathesis and ethenolysis of methyl oleate compared to Grubbs first generation ruthenium catalyst **1**. Previously the Sasol group reported air-stable and recyclable ruthenium-based catalysts [(PhobCy)_2_Cl_2_Ru=CHC=C(Me)_2_]] (**5**) and [(PhobCy)_2_Cl_2_Ru=CHPh] (**6**) which can be used in various metathesis reactions [57]. Recently, Thurier *et al.* [58] reported the ethenolysis of methyl oleate using ruthenium alkylidene catalysts (**1, 3, 7**–**11**) in toluene and in room temperature ionic liquids. Complexes **10** and **11** are Hoveyda-type catalysts bearing an ionic imidazolium branch. The results in toluene showed that **7** (first generation Hoveyda-Grubbs catalyst) had better efficiency and selectivity than the rest of the catalysts with 91% conversion of the methyl ester. Furthermore, **7** of all the catalysts used, could be recycled a number of times in ionic liquids without a dramatic loss of activity. This is yet another positive achievement bringing the commercialization of the metathesis of vegetable oils closer to reality. Ruthenium complexes **1**–**11** are illustrated in Figure 1.

### 3.3. α-Olefins

**Scheme 5:**
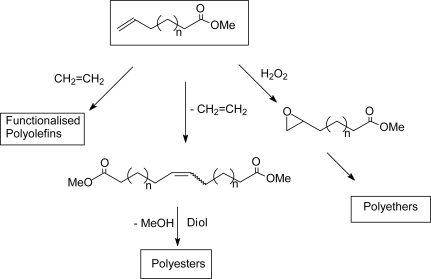
Ethenolysis of triolein.

α-Olefins can be produced from internally unsaturated fatty acid esters by cross-metathesis with ethene. In scheme 3, for example, 1-decene is produced by ethenolysis of oleic acid methyl ester; an example of the conversion of low value olefins to high value olefins [8]. α-Olefins have numerous applications, for example, in the formulation of detergent alcohols, plasticizer alcohols, poly(α-olefins), epoxides, alkyl aromatics, personal care products, flavors and fragrances [59]. Treatment of α-olefins with peracids produces epoxides which find application in the formulation of epoxy resins, polyethers and polyurethanes. Ethenolysis of triolein (main constituent in high oleic sunflower oil) also yields 1-decene and glyceryl tridec-9-enoate as shown in Scheme 5 [60]. Glyceryl tridec-9-enoate is a source of methyl 9-decenoate, which is an important starting material in the synthesis of a wide variety of polymers. It could also be converted to tricaprin by hydrogenation [61]. Tricaprin is used in personal care products and cosmetics. Ethenolysis of linoleic acid ester (from regular sunflower oil), on the other hand, results in 1-heptene, 1,4-pentadiene, 1,4-decadiene, methyl 9-decenoate and methyl 9,12-tridecadienoate [61].

Several catalysts based on W, Re and Ru have been successfully applied in processes leading to the production of α-olefins. However, the well-defined Ru-based catalysts are preferred due to their stability and exceptional tolerance for functional groups and oxygenates. The formulation of α-olefins from renewable resources *via* olefin metathesis offers an alternative and sustainable route to the currently used Fisher-Tropsch process which utilizes synthesis gas from coal or natural gas [62].

### 3.4. Fragrances

**Scheme 6:**
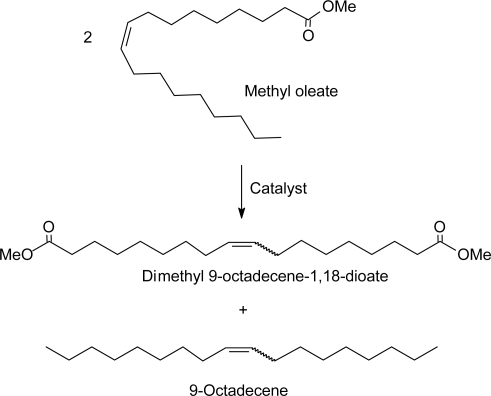
Ring closing metathesis of oleon.

Flavor and fragrance compounds with aroma are present in nature in many living organisms such as flowers, fruits, nuts and animals and range from terpenes to phenols and from aldehydes to esters [63]. The flavors and fragrances form part of the global specialty chemicals industry and make it possible for certain flavors and fragrances to be added to a huge range of products [64]. Flavor and fragrance compounds can be obtained by (1) isolation from natural sources (plants and meat) (2) chemical and biochemical synthesis or (3) by fermentation [63]. Olefin metathesis has also proved useful in the formulation of the starting materials for some of the fragrances. For example, neohexene, a starting material for polycyclic musks such as Tonalide®, is industrially obtained by cross metathesis of 2,4,4-trimethyl-2-pentene with ethene [62]. In addition, unsaturated dicarboxylic acid esters can be synthesized by self-metathesis of oleic acid esters (as shown in Scheme 4) using WOCl_4_/Cp_2_TiMe_2_ catalyst and subsequently converted to macrocyclic compounds such as civetone, an important ingredient in musk perfumes [65]. A convenient new synthetic route to civetone was also demonstrated in which methyl oleate (from high oleic sunflower oil) is first converted *via* a Claisen condensation reaction to oleon (9,26-pentatriacontadien-18-one). The latter is subsequently converted to civetone by a ring closing metathesis reaction (Scheme 6) using Re_2_O_7_/SiO_2_−Al_2_O_3_/Bu_4_Sn catalyst [60]. Another metathesis-based route to civetone involves dimerization of methyl 9-decenoate to form 1,18-nonadecadien-10-one, followed by ring-closing metathesis [61].

### 3.5. Challenges and Future Perspectives

Although significant developments in catalyst design and performance have been achieved, generally low turnovers and catalyst stabilities still limit commercial application of the processes involving seed oil based feedstocks. For the ethenolysis of methyl oleate, for example, turnovers exceeding 50 000 are to be achieved for an economically viable process, according to the group at Dow [41]. Concerted research efforts are, therefore, still needed in order to gain a detailed understanding of the factors which impact catalyst performance and possibly even new approaches that would result in economically viable processes. Competing reactions such as isomerisation also pose a challenge in that lower yields of targeted product(s) are obtained, thus making product separation a cumbersome exercise. Nevertheless, promising results with very high turnover numbers and selectivity have been reported for some ruthenium-based catalysts [53, 56, 58, 66], some of which could even be recycled without the drastic loss of activity. These results represent an achievement which brings the commercialization of the metathesis of vegetable oils closer to reality.

On the other hand, price and limited supply have led to sunflower oil being used mostly as edible oil. However, continued improvements in oil quality and yield as well as the soaring prices of crude oil are likely to push its demand higher for nonfood applications thus making it more competitive to justify more land for cultivation. Ongoing discussions are also needed between researchers, industry leaders and policy makers in order to gain a common understanding around issues of oil performance requirements, supply chains and food security. With regard to biodiesel market, reliable standards are still to be developed in order to instill confidence in biodiesel users and engine manufacturers, points out Knothe *et al.* [33]. A breakthrough on the challenges highlighted should hopefully lead to full scale commercialization of some or most of the processes so far discussed.

## 4. Summary

Price and limited supply have led to sunflower oil being used mostly for human consumption. However, the soaring prices of crude oil and continued improvements in oil quality and yield are likely to push its demand higher to justify more acres of cultivated land. On the other hand, recent improvements in catalyst performance offer promising new opportunities, especially in biofuel, biopolymers, fine chemicals and fragrance applications. The possibilities in nonfood applications present a promising future to sunflower-based feedstocks in the global world market. Finally, concerted research efforts and ongoing discussions between researchers, industry leaders and policy makers are critical in making the commercialization of seed oil feedstocks a reality.

## Figures and Tables

**Table 1:** Properties of selected biodiesel fuels a.

Entry	Biodiesel^b^	CN^c^	CP^d^ (ºC)	PP^e^ (ºC)	FP^f^ (ºC)	Viscosity^g^ (mm^2^/s)
1	Sunflower oil	46.6	0	−4	-	4.22
2	Safflower	49.8	-	−6	180	-
3	Soybean	46.2	2	−1	171	4.08
4	Rapeseed	54.4	−2	−9	84	6.7
5	Palm	56.2	8	6	19	4.5

^a^ Ref. [33]

^b^ Methyl esters (entries 1–4), Ethyl ester (entry 5)

^c^ CN (Cetane Number)

^d^ CP (Cloud Point)

^e^ PP (Pour Point)

^f^ FP (Flash Point)

^g^ Measured at 40ºC (entries 1–4) and at 37.8ºC (entry 5)

**Table 2:** Biodiesel composition and conversion *via* olefin metathesis.

Source	Biodiesel	Composition (%)	Conversion (%)	Ref
Sunflower	Methyl esters^a^		Re_2_O_7_^d^	35
(Regular)	C16:0	7.3	−	
	C18:0	6.0	−	
	C18:1	20.6	81 (3h)	
	C18:2	65.1	45 (3h)	
	C20:0	0.3	−	
	C22:0	0.7	−	

Sunflower	Ethyl esters^b^		WCl^e^_6_	34
(Regular)	C16:0	7.0	−	
	C18:0	6.0	−	
	C18:1	28.0	84 (3h)	
	C18:2	59.0	50 (3h)	

Soya	Methyl esters^c^		Ru^f^	36
	C16:0	11.0	−	
	C18:0	5.0	−	
	C18:1	23.0	30 (2h)	
	C18:2	54.0	50 (2h)	
	C18:3	7.0	70 (2h)	

^a^ Obtained by direct transesterification of sunflower oil from Continental Oil Mills (South Africa),

^b^ Derived from South African sunflower oil;

^c^ Methyl soyate obtained from Soygold (USA),

^d^ 3% Re_2_O_7_/SiO_2_−Al_2_O_3_/SnBu_4_ at 20ºC (0.2g catalyst and 0.5 ml substrate);

^e^ WCl_6_/SnMe_4_ at 110–120ºC (1.4g catalyst and 40 mL substrate);

^f^ RuCl_2_(PCy_3_)(H_2_IMes)(=CHPh) at 40ºC (10g substrate and 0.1wt% catalyst)
